# Cancer survivors’ adherence to the American cancer society and American institute of cancer research dietary guidelines in Lebanon

**DOI:** 10.1186/s12889-024-20099-3

**Published:** 2024-10-08

**Authors:** Jana Jabbour, Remie El Helou, Ruba Hadla, Riwa Azar, Maria Mezher, Farah Naja, Sally Temraz

**Affiliations:** 1 Nutrition Program, Department of Natural Sciences, Lebanese American University, Beirut, Lebanon; 2 Department of Internal Medicine, American University of Beirut Medical Center, Beirut, Lebanon; 3 Clinical Nutrition and Dietetics Department, College of Health Sciences, University of Sharjah, Sharjah, UAE

**Keywords:** Cancer survivors, Remission, Diet, Physical activity, Overweight, Whole grains, Compliance

## Abstract

**Background:**

Adequate diet and lifestyle practices are postulated to improve health and enhance wellbeing of cancer survivors. Despite the heavy cancer burden in Lebanon, little is known about the diet quality of survivors. This cross-sectional study assessed the compliance of survivors in remission with the American Cancer Society/American Institute Research Fund (ACS/AICR) diet and physical activity guidelines.

**Methods:**

Cancer survivors in remission for at least 3 months and attending oncology clinics in two referral centers in Beirut, Lebanon were recruited. Adherence to the ACS/AICR was assessed by examining the compliance to guidelines promoting fruits, vegetables and whole grain and discouraging elevated alcohol, meat and energy dense foods intake. Dietary data was assessed through food frequency and lifestyle questionnaires administered face to face and through phone interviews. Anthropometrics, food security and sociodemographic data was also collected. Univariate and bivariate descriptive and logistic regressions were used to determine predictors of adherence rates to the ACS/AICR score.

**Results:**

A total of 268 participants were recruited (mean age = 59 ± 23 years, 83% females, 70% breast cancer). Mean time since remission was 3.2 ± 4 years and the majority (72%) had food insecurity. Low physical activity and overweight/obesity were present in 65% and 67% of the survivors, respectively. Median adherence score with the ACS/AICR score was 9.0 out of 15. The majority of survivors had complete adherence to the moderate meat and alcohol consumption guidelines. However, 98% were noncompliant with the whole grains’ consumption guidelines. Cancer type, site of recruitment and education were the significant predictors of the ACS/AICR diet adherence score.

**Conclusion:**

This study highlights the elevated rates of overweight/obesity, physical inactivity and the alarmingly low adherence with the whole grains consumption guideline among cancer survivors in remission. Policy makers ought to use study findings to redirect government subsidization and educational interventions in the country and physicians should stress the importance of adherence to a healthy diet during remission through counseling and timely referral.

**Supplementary Information:**

The online version contains supplementary material available at 10.1186/s12889-024-20099-3.

## Background

Cancer survivors are individuals who have been diagnosed with cancer and are on treatment, in remission, or receiving palliative care [1]. Compared with persons who have not had cancer, survivors are at greater risk for developing secondary malignancies, cardiovascular diseases, diabetes, osteoporosis, and functional decline [2–7]. Several research bodies have issued diet and physical activity (PA) recommendations to guide health care providers in shaping the nutrition and PA regimens of cancer survivors [1, 8, 9]. The World Cancer Research Fund/American Institute of Cancer Research (WCRF/AICR) and American Cancer Society’s (ACS) guidelines are among the most cited references for cancer patients in remission [1, 8, 10]. These guidelines promote PA, breastfeeding, the regular consumption of fruits, vegetables and wholegrains, limiting the consumption of alcohol and advise against the elevated intake of red and processed meat and added sugars [1, 8, 9].

Lebanon has been characterized by following the Mediterranean diet [11]. Yet, there has been a gradual decline in the adherence of Lebanese to the latter diet due to the adoption of westernized dietary patterns and the rise in food insecurity [11–13]. Lebanon has a heavy cancer burden compared to other countries in the region [14–16] and cancer incidence, associated with dietary and PA factors, has been on the rise in the past decades [17–19]. Despite this, the evidence on the reduced morbidity and mortality associated with adherence to diet and PA guidelines for cancer survivors is lacking, little is known about the current diet and PA status of survivors in Lebanon and the Middle East and North Africa Region (MENA) [20, 21]. To address this gap, this study aims to assess the adherence of cancer survivors in Lebanon with the ACS/AICR diet and PA guidelines with a main focus on dietary components.

## Methods

This cross-sectional study follows the STROBE guidelines for observational studies (STROBE statement available in Table S1) [22]. Data collection was performed between August 2018 and February 2022 at the American University of Beirut Medical Center (AUBMC) and at Makassed General Hospital (MGH) in Beirut, Lebanon. These medical centers were chosen as they are considered major referral centers and serve patients from different socioeconomic backgrounds across Lebanon and the region. The study was conducted in compliance with the ethical principles of the declaration of Helsinki and the Belmont report. The research project received approvals from the Institutional Review Board of AUBMC and the Institutional Review Board of MGH before the initiation of any research-related activity. Patients attending AUBMC and MGH’s private clinics and dispensaries were approached by members of the research team who informed them of the study goals, benefits, and risks as well as their right to withdraw from the study at any point during the interview without any repercussion on the quality of care received. Those who were interested and available to enroll in the study provided written and oral consent to participate.

Adult patients (≥ 18 years) previously diagnosed with one of the eight most prevalent cancers in Lebanon [breast, respiratory, colorectal, prostate, lymphoma (“Hodgkin and Non-Hodgkin”), bladder, stomach, and leukemia] according to the National Registry Data and in remission at the time of study conduct were included in the study [23]. Remission was ascertained using the report of the last Computed Tomography and/or the oncologist’s note on the patients’ medical charts. Exclusion criteria included having terminal illness, being on palliative care and/or having visual or cognitive impairment.

### Assessment tools

Participants were asked to report on lifestyle questions related to their education, socioeconomic status (SES), occupation and living arrangement. The crowding index (CI) was calculated by dividing the number of household rooms, excluding bathrooms, balconies, and kitchens, by the number of people living in a household. CI > 1 reflected crowded households [24]. Participants were asked to report on their monthly household incomes in Lebanese Liras. Food insecurity was evaluated using the Arab Family Food Security Scale, a tool that has been validated in Lebanon [25]. Participants with scores ≤ 2, 3–4 and ≥ 5 were considered to have food security, moderate food insecurity and severe food insecurity, respectively [25].

Subjects’ PA was evaluated using the International Physical Activity Questionnaire- short form (IPAQ- SF). This tool, validated against accelerometers [26], has been adapted to several languages including the Arabic language [27]. This questionnaire includes 9 questions about the frequency of having vigorous and moderate PA as well as duration spent walking and sitting. The scores allow for the classification of individuals as having low, moderate and elevated physical activity based on the intensity and duration of PA [27]. Participants’ weight and height were collected from the medical records of each facility.

Dietary intake was assessed using a 119 item Food Frequency Questionnaire (FFQ) adapted from a validated one in Lebanon [28]. FFQ was administered by trained dietitians who asked participants to report the number of exchanges as well as the frequency (daily, weekly, monthly, rarely/never) of consumption of food items commonly consumed in Lebanon. Portion sizes were explained in common household measurements (e.g. tablespoons, cups) to facilitate estimation. Nutrient analysis of the FFQ was done by incorporating the food composition of consumed food and beverages on Microsoft Excel (Microsoft Corporation, Washington, 2016). Food composition of FFQ items was derived from locally specific resources [29–31] as well as from FoodData Central, a database by the United States’ Department of Agriculture [32]. Adherence to the ACS/AICR guidelines was assessed using a score that was operationalized by Springfield et al. [33]. We opted to use the ACS/AICR scoring method over the WCRF/AICR score since data on waist circumference and breastfeeding were not available in our cohort. The score (available in Table S2) favored intake of vegetables and fruits as well as whole grains and opposed elevated intake of red and processed meat, energy dense foods and alcoholic beverages. Participants were categorized into one of the following groups for each of the dietary component assessed: 0: non adherent, 1: modestly adherent, 2: moderately adherent and 3: completely adherent. Subjects who had scores above and equal to the median were considered to have elevated adherence (EA) and those below the median level were classified as having low adherence (LA).

Patients’ medical charts were reviewed to extract information on the following variables: Last weight measurement, height, date of birth, nationality, cancer related variables (cancer type and cancer stage, date of recurrence and of remission, chemotherapy regimens received, stem cell transplantation received) and comorbidities (e.g. chronic kidney disease, diabetes mellitus, hypertension). Charlson comorbidity index (CCI) was used to assess the burden of comorbidities on the participants’ health, with higher scores reflecting a greater weight of comorbidities [34, 35].

### Changes due to the COVID 19 pandemic

The study was launched prior to the onset of the COVID 19 pandemic and continued thereafter. In March 2020, the IRB of both medical centers requested that researchers withhold all activities that involved live encounters with patients to minimize the spread of COVID 19. Research activities were suspended at both sites. In July 2020, the IRB of AUB allowed research activities to be resumed by telephone assessment. As it was possible to collect data by telephone, interviews with subjects were shifted to phone assessments. Members of the research team were informed by the medical and nursing teams of eligible participants who were interested to enroll in the study. The research team contacted them by phone thereafter to introduce the study protocol and collected data from those willing to participate after obtaining oral consent. Data collection was not resumed in MGH as the suspension of research activities was further delayed at this medical center.

### Research team

The research team included an oncologist as well as dietitians and researchers in the field of oncology and nutritional epidemiology. To minimize inter-rater variability, the co-principal investigator, an experienced dietitian, provided training based on written standards of operations for live and telephone assessments.

### Sample size and statistical considerations

Cohort and cross-sectional studies assessing adherence of cancer survivors have found adherence rates ranging between 12% and 49% for PA and between 15% and 19% for diet guidelines [36, 37]. Accordingly, it was hypothesized that the sample would have a 25% compliance rate with PA and diet guidelines. The number of incident cases of the most prevalent cancers was 7,356 in year 2015, the most recent national statistic at the time of proposal writing [23]. Assuming an estimated rate of 50% of survivorship, a power of 80% and a 5% margin error, the sample size to assess compliance in diet and PA guidelines was 268 participants.

Chi-square and independent-t tests were employed to compare the categorical and continuous variables, respectively, across adherence groups. Univariate and multivariate logistic regression using the enter method were used to assess predictors of high adherence. Multicollinearity was identified when the correlations coefficient or the Variance Inflation Factors (VIF) exceeded 0.7 and 10, respectively. Statistical significance was set at the conventional level of *p* < 0.05. Since data was not missing neither for the outcomes of interest nor for the variables entered in the regression model, missing data was not imputed and was presented as such. Measures of analyses were performed on IBM SPSS version 25 (SPSS Inc. Chicago, IL, USA).

## Results

A total of 268 cancer survivors were recruited in this study (Table 1). Main reasons for not participating in the study were lack of time and/or interest. Participants had a mean age 59 ± 23 years. Data was not missing for any of the outcomes assessed. Most of the participants were females (83%), living with others and in urban areas and holding a university degree. Half of the cancer survivors lived in crowded homes and most of the sample (71%) had severe food insecurity. The majority of the sample (94%) was recruited from AUBMC (Table 1).

**Table 1:** Participants’ sociodemographic characteristics (*n* = 268)

Characteristic	
Age (years), mean ± SD	59 ± 23
Females, *n* (%)	222 (83)
Living arrangements, *n* (%)	
Living alone	18 (7)
Living with others	250 (93)
Area of residence, *n* (%)	
Urban	157 (59)
Sub-urban	65 (24)
Rural	46 (17)
Crowding Index, *n* (%)	
< 1	134 (50)
≥ 1	132 (50)
Educational level, *n* (%)	
Illiterate	6 (2)
Primary school	45 (17)
High school	84 (31)
University	132 (49)
Site of recruitment, *n* (%)	
Site 1 (AUBMC)	251 (94)
Site 2 (MGH)	17 (6)
Face to face recruitment, *n* (%)	162 (60)
Food security, *n* (%)	
Secure	75 (28)
Moderately insecure	4 (1)
Severely insecure	189 (71)

AUBMC: American University of Beirut Medical Center, MGH: Makassed General Hospital

Table 2 features the health-related characteristics of the participants. Breast cancer was the most common cancer, followed by hematological and gastrointestinal (GI) cancers. Most of the participants (79%) had early stage tumors and only 6% had had a prior relapse. The mean time since remission was 3.2 ± 4 years. Survivors had a mean CCI of 1.7 ± 1 and a mean Karnofsky performance score of 93 ± 8.4. The majority of participants were overweight or obese (67%) and had a low physical activity profile (65%), with a mean daily sitting time of around 6 h. Only 23% received prior education on diet and/or PA (Table 2), 14% of the survivors consulted a dietitian, 3% received referral from their physicians for dietetic counseling and 6% self-referred themselves to a dietitian (data not shown). Finally, with regards to smoking, 30% of participants reported being daily or occasional smokers (Table 2).

**Table 2:** Participants’ health related characteristics

Primary cancer group, *n* (%)	
Breast	187 (70)
Hematology	40 (15)
Gastrointestinal	22 (8)
Respiratory	7 (3)
Others	12 (5)
Tumor stage, *n* (%)	
≤ 1	105 (46)
2	74 (33)
3	30 (13)
4	12 (5)
Unstaged	6 (3)
Prior relapse, *n* (%)	15 (6)
Time since remission (years), mean ± SD	3.2 ± 4
Charlson comorbidity index, mean ± SD	1.7 ± 1
Karnofsky performance status, mean ± SD	93 ± 8.4
Body mass index category, *n* (%)	
Underweight	2 (0.8)
Normal	79 (33)
Overweight or obese	162 (67)
Received education on diet and/or physical activity from any health professional, *n* (%)	65 (24)
Physical Activity Level, *n* (%)	
Low	174 (65)
Moderate	66 (25)
High	28 (10)
Daily sitting time (hours), mean ± SD	5.9 ± 3
Smoking, *n* (%)	
Daily smokers	59 (23)
Occasional smokers	19 (7)
Ex-smokers	38 (15)
Non smoker	146 (56)

**Fig. 1:**
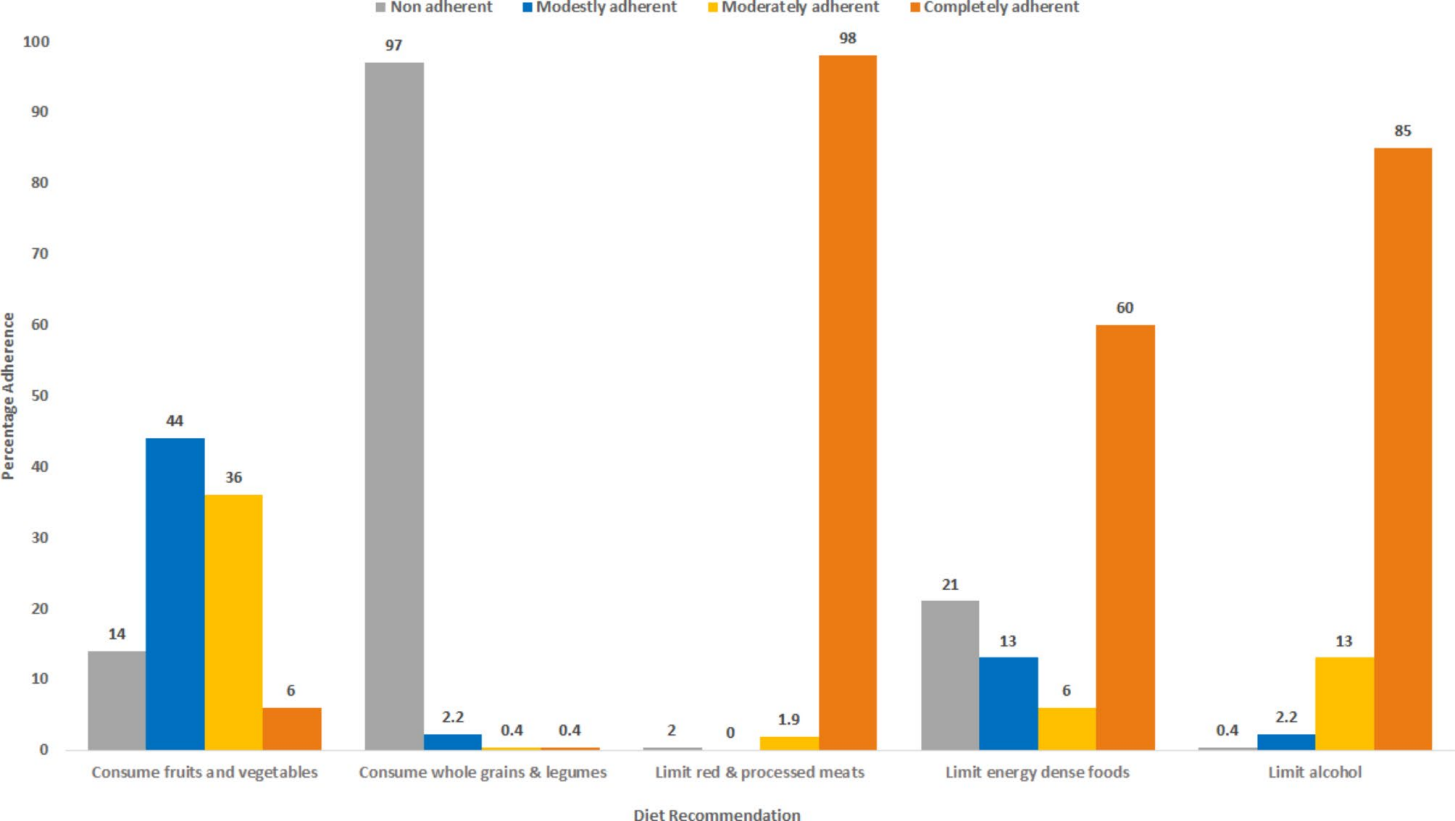
The relative adherance by cancer survivors to the dietary guidelines set by The American Cancer Society/American Institute of Cancer Research (ACS/AICR). Participants were categorized in one of the following groups for each of the dietary component assessed: 0: non adherent, 1: modestly adherent, 2: moderately adherent and 3: completely adherent

Assessed cancer survivors had a moderate adherence to fruits and vegetables recommendation, with only 6% of them regularly consuming 5 or more cups per day (Fig. 1). Adherence was very low for the recommendation of consuming whole grains with 97% of participants having an adherence score of zero for this guideline. In contrast, participants had an elevated adherence to limiting red and processed meat (98%) and alcohol consumption (85%). Adherence to regular consumption of energy dense items was acceptable with 60% of participants having an elevated adherence score (Fig. 1). The median adherence score to the ACS/AICR was 9.0. Table 3 presents the characteristics of participants that that had a low (*n* = 131) and a high adherence rate (*n* = 138) in relevance to the median score. Participants with a low adherence rate were more likely to be female, to have a university level education, to be food insecure, and to have hematological or GI cancer (Table 3).

**Table 3:** Comparison of the characteristics by median adherence ACS/AICR score

Characteristic	Low adherence (*n* = 131)	Elevated adherence (*n* = 138)	*P* value
Age (years), mean ± SD	58 ± 12	59 ± 29	0.527
Female, *n* (%)	98 (75)	124 (90)	< 0.01
Living arrangements, *n* (%)			0.896
Living alone	9 (6.9)	9 (6.5)	
Living with others	121 (93)	129 (94)	
Area of residence, *n* (%)			0.119
Urban	69 (53)	88 (64)	
Suburban	33 (25)	32 (23)	
Rural	28 (22)	18 (13)	
Crowding Index, *n* (%)			0.464
< 1	62 (48)	72 (53)	
≥ 1	67 (52)	65 (47)	
Educational level, *n* (%)			0.022
Illiterate	1 (0.8)	5 (3.6)	
Primary school	15 (12)	30 (22)	
Secondary school	40 (31)	44 (32)	
University level	74 (57)	58 (42)	
Site of recruitment, *n* (%)			0.02
Site 1 (AUBMC)	128 (99)	123 (89)	
Site 2 (MGH)	2 (1.5)	15 (11)	
Face to face recruitment, *n* (%)	73 (56)	89 (65)	0.163
Food security, *n* (%)			0.142
Secure	43 (33)	32 (23)	
Moderately insecure	1 (0.8)	3 (2.2)	
Severely insecure	86 (66)	103 (75)	
Cancer type, *n* (%)			0.03
Breast	76 (59)	111 (80)	
Hematology	26 (20)	14 (10)	
Gastrointestinal	16 (12.4)	6 (4)	
Respiratory	5 (4)	2 (1.4)	
Others	6 (4.7)	5 (3.6)	
Time since remission, mean ± SD	3.5 ± 4.8	2.9 ± 3.4	0.372
Received education on diet and/or physical activity from any health professional, *n* (%)	36 (28)	26 (19)	0.081

AUBMC: American University of Beirut Medical Center, MGH: Makassed General Hospital

Table 4 features univariate and multivariate logistic regression of poor adherence to the ACS/AICR dietary guidelines. At the univariate stage, gender, area of residence, education level, recruitment site, food security, cancer type and prior education on diet and PA guidelines had *p* values below 0.15 and entered in the regression model. At the multivariate level, three variables remained significant: Higher education, site of recruitment and cancer type. Survivors with a university level education had a higher risk of having a poor adherence [OR (95%CI):1.8 (1.06–3.05)] whereas those recruited at MGH [OR (95%CI):0.060 (0.01–0.6)] and with a history of breast cancer had reduced chances of poor adherence to the ACS/AICR dietary guidelines compared to those recruited at AUBMC and those having other types of cancers.

**Table 4:** Univariate and multivariate logistic regression of the predictors of a low ACS adherence score

Characteristic	Univariate analysis			Multivariate analysis		
	OR	95% CI	*P* value	OR	95% CI	*P* value
Age (years)	0.996	0.984–1.01	0.539			
Female sex	2.89	1.46–2.89	0.02	1.220	0.45–3.37	0.695
Suburban/rural area of residence	1.83	0.96–3.50	0.068	1.980	1.00- 3.95	0.051
Higher education	1.80	1.11–2.92	-	1.790	1.06–3.05	0.031
Site 2 (MGH)	0.128	0.029–0.572	< 0.01	0.090	0.01–0.74	0.025
Telephone assessment	1.42	0.87–2.32	0.164			
Food insecure	0.611	0.357–1.05	0.073	0.680	0.38–1.22	0.194
Breast Cancer	0.349	0.202–0.603	< 0.01	0.388	0.177–0.854	0.019
Time since remission (years)	1.04	0.957–1.13	0.358			
Received education on diet and/or physical activity from any health professional	1.67	0.940–2.97	0.080	1.65	0.890–3.07	0.112

MGH: Makassed General Hospital. Variables assessed: age (years), gender (female vs. male), area of residence (suburban/rural vs. urban), education (higher education vs. others), food security (moderate/severe insecure vs. food secure), cancer type (breast vs. others), time since remission (years), and receiving health education (yes vs. no)

## Discussion

Compliance with the ACS/ AICR recommendations has been associated with improved quality of life, mortality and reduced cancer incidence [38–40]. This study is the first in Lebanon and the Middle East region to assess the prevalence and the predictors of the adherence to the ACS/AICR guidelines among individuals in cancer remission. Results revealed that around two third of participants had a low level of physical activity and were either overweight or obese. Adherence to the ACS recommendations was elevated for the meat and alcohol consumption guideline and was low for the consumption of legumes and whole grains. Having breast cancer and a lower level of education were independent predictors of elevated adherence.

Proper weight management, having a normal BMI and active engagement in physical activity are among the ACS/AICR pillars when discussing diet and PA recommendations for cancer .survivors. Yet, 67% of cancer survivors in this study had excessive weight and 65% had a low activity profile. Of interest, only 3% of physicians referred patients for dietetic counseling and 76% of survivors did not receive any kind of education on diet and physical activity. A previous cross-sectional study in a sample of individuals with diabetes type 2 in Lebanon revealed that physicians’ referral greatly affected the chances that individuals consult a dietitian as patients greatly valued their doctors’ recommendations [41]. Despite the elevated prevalence of malnutrition among individuals with cancer, access to dietetic counseling has been a worldwide concern [42, 43]. A national cross-sectional study in Ireland revealed that 57% of cancer patients were not assessed by a Registered Dietitian (RD) even though they welcomed and needed dietetic support [44]. A qualitative study from the United Kingdom emphasized patients’ expressed need for proper and timely dietetic guidance [42] and an online assessment of cancer survivors revealed that 42% sought nutritional advice from any health care provider [43]. In our study, receiving education on diet and physical activity from any health care professional was not an independent predictor of increased adherence to the ACS/AICR score, possibly because this variable reflected any kind of advice received from any health professional irrespective of its relevance, accuracy and timing. An integrative review on the topic revealed that allied health professionals and nurses were unsure of their role in referring patients to dietetic and PA counseling and did not consider themselves competent to provide such advice [45].

Adherence to the guideline of consuming fruits and vegetable was expected to be high in a country such as Lebanon known for following the Mediterranean diet [46]. Yet, around 58% of the sample had a low adherence rate to this guideline. The reduced consumption of fruits and vegetables can be explained by the nutritional transition the country is going through and the increased food insecurity residents are experiencing [47, 48]. Indeed, residents across the lifespan have been shifting towards a westernized diet, associated with a greater consumption of sodium and energy dense foods and a reduced intake of fruits and vegetables [49, 50]. This shift has been associated with a reduced diet quality and greater odds of developing obesity and the metabolic syndrome [51–53]. Rates of adherence to the fruits and vegetable guidelines are comparable to results found in a systematic review on the topic [54].

A similar cross-sectional study assessed adherence rates of African American breast cancer survivors with the ACS/AICR guidelines [33]. Both studies found an elevated adherence to the moderate meat consumption and limited adherence to the fruits, vegetables and whole grains consumption. Complete adherence with the red and processed meat (98% vs. 78–88%) and alcohol consumption (85% vs. 37%) were greater in our study. Yet, adherence with the whole grains consumption was more alarming in this study with 98% of survivors having an adherence score of zero compared to 66% of participants in the Springfield et al. study [33]. Our findings are also more concerning than those from a recent online survey based on participants’ self-evaluation which revealed that adequate whole grain consumption was the guideline with the lowest adherence rate with around 62% of survivors not complying to it [43].

This study identified cancer type, education level and site of recruitment to be significant predictors of adherence rate in multivariate regression. Individuals with breast cancer had a higher adherence rate than those with GI cancers. This finding calls for more interventions to target individuals with a history of cancer other than breast, especially GI cancers. Education is known to be a positive predictor of adherence to dietary guidelines [33]. Paradoxically, educational level was a negative predictor of compliance rate with cancer survivors. We are unable to explain the underlying reason for this association, especially that neither quantitative data on employment rate nor qualitative data on barriers for compliance were collected. Future studies should assess this association and explore relevant underlying reasons. Site of recruitment was the third significant predictor of adherence rate. Participants recruited at AUBMC had a lower likelihood of adhering to the dietary ACS/ AICR guidelines. It is not clear how recruitment site could have affected adherence rates. This result may have been influenced by the small number of subjects recruited at MGH. Future studies should further investigate the effect of recruitment site and the associated demographic factors on adherence rate.

Due to the compounded economic, political, environmental and health systems disasters, Lebanon has been considered on the verge of collapse [55–57]. In 2019, Lebanon was hit by a still ongoing economic crisis, considered among the top ten crises since the 19th century [58]. In 2020, the country was the host of the third largest non-nuclear blast in the world history [59]. The rate of severe food insecurity in medical centers known to serve different SES identified in our study is alarming. This can partially explain the low level of adherence to the ACS/AICR recommendations for whole grains consumption, the elevated adherence with the consumption of processed and red meat and alcohol. Indeed, whole wheat bread is more expensive than white bread as the former is not subsidized by the government and is not as available as white bread [60]. Similarly, red and processed meat and alcohol are considered expensive and less accessible than other food components in times of food insecurity [61].

This study has several strengths and limitations. It employs a solid methodology using questionnaires validated and/or adapted to the population assessed and implemented by licensed dietitians. It takes into consideration anthropometric, dietary, and clinical data and is the first in the region to answer this research question. Limitations of this study relate to the population and timing of assessment. Like the rest of the world, Lebanon had to impose strict public health measures limiting physical contact during the COVID-19 pandemic [62]. Our study was started before the pandemic and continued thereafter. The public health measures imposed in 2019 to limit the spread of COVID 19 shifted the mode of data collection from live to telephone assessment. To evaluate the difference in administration technique, telephone vs. face to face recruitment were assessed through bivariate and univariate regression. In terms of generalizability, results of this study apply to individuals in cancer remission seeking care in Beirut, Lebanon. Recruiting participants from medical centers across the country would have provided results that can be more generalizable.

## Conclusion

To our knowledge, this is the first study assessing the adherence of cancer survivors in Lebanon with the ACS/AICR guidelines. It revealed that overweight/obesity and physical inactivity rates were elevated, adherence rate to the whole grains consumption guideline is alarmingly low and compliance with the fruits and vegetables and limited energy dense items recommendations was moderate. These findings call for action by policy makers to subsidize whole wheat bread and for food manufacturers to reformulate their products to increase the proportion of whole grains in Lebanon. In view of the higher adherence rate to dietary guidelines among breast cancer survivors, multimodal interventions should prioritize GI cancers and other cancer types over breast cancer at this point, when limited resources are available. At the level of the multidisciplinary team, this study identifies the need for oncologists to timely refer their patients when in remission to consult a dietitian and/or behavioral therapist to optimize their lifestyle. Future studies should validate the findings among individuals from different regions of Lebanon and investigate the effect of education and employment on adherence rate.

## Electronic supplementary material

Below is the link to the electronic supplementary material.

Supplementary Material 1

## Data Availability

The datasets used and/or analyzed during the current study are available from the corresponding author on reasonable request.
