# Supplementary material for: Cancer survivors’ adherence to the American cancer society and American institute of cancer research dietary guidelines in Lebanon
Source: BMC Public Health. 2024 Oct 8;24:2738. doi: 10.1186/s12889-024-20099-3 (PMC11462921; doi:10.1186/s12889-024-20099-3)
Supplement: Supplementary file 1 — Supplementary Material 1 [file 12889_2024_20099_MOESM1_ESM.docx]

Table S1: STROBE Statement—Checklist of items that should be included in reports of cross-sectional studies

|  | Item No | Recommendation | Page No |
| --- | --- | --- | --- |
| **Title and abstract** | 1 | (*a*) Indicate the study’s design with a commonly used term in the title or the abstract | 1-2 |
|  |  | (*b*) Provide in the abstract an informative and balanced summary of what was done and what was found | 2 |
| Introduction | | | |
| Background/rationale | 2 | Explain the scientific background and rationale for the investigation being reported | 3 |
| Objectives | 3 | State specific objectives, including any prespecified hypotheses | 3 |
| Methods | | | |
| Study design | 4 | Present key elements of study design early in the paper | 4 |
| Setting | 5 | Describe the setting, locations, and relevant dates, including periods of recruitment, exposure, follow-up, and data collection | 3-4 |
| Participants | 6 | (*a*) Give the eligibility criteria, and the sources and methods of selection of participants | 4 |
| Variables | 7 | Clearly define all outcomes, exposures, predictors, potential confounders, and effect modifiers. Give diagnostic criteria, if applicable | 4-5 |
| Data sources/ measurement | 8 | For each variable of interest, give sources of data and details of methods of assessment (measurement). Describe comparability of assessment methods if there is more than one group | 4-5 |
| Bias | 9 | Describe any efforts to address potential sources of bias | 6 |
| Study size | 10 | Explain how the study size was arrived at | 6 |
| Quantitative variables | 11 | Explain how quantitative variables were handled in the analyses. If applicable, describe which groupings were chosen and why | 6-7 |
| Statistical methods | 12 | (*a*) Describe all statistical methods, including those used to control for confounding | 6-7 |
|  |  | (*b*) Describe any methods used to examine subgroups and interactions | 6-7 |
|  |  | (*c*) Explain how missing data were addressed | 7 |
|  |  | (*d*) If applicable, describe analytical methods taking account of sampling strategy | N/A |
|  |  | (*e*) Describe any sensitivity analyses | N/A |
| Results | | | |
| Participants | 13 | (a) Report numbers of individuals at each stage of study—eg numbers potentially eligible, examined for eligibility, confirmed eligible, included in the study, completing follow-up, and analysed | 7 |
|  |  | (b) Give reasons for non-participation at each stage | 7 |
|  |  | (c) Consider use of a flow diagram | N/A |
| Descriptive data | 14 | (a) Give characteristics of study participants (eg demographic, clinical, social) and information on exposures and potential confounders | 7 |
|  |  | (b) Indicate number of participants with missing data for each variable of interest | Table 2 |
| Outcome data | 15 | Report numbers of outcome events or summary measures | N/A |
| Main results | 16 | (*a*) Give unadjusted estimates and, if applicable, confounder-adjusted estimates and their precision (eg, 95% confidence interval). Make clear which confounders were adjusted for and why they were included | 7-8 |
|  |  | (*b*) Report category boundaries when continuous variables were categorized | N/A |
|  |  | (*c*) If relevant, consider translating estimates of relative risk into absolute risk for a meaningful time period | N/A |
| Other analyses | 17 | Report other analyses done—eg analyses of subgroups and interactions, and sensitivity analyses | N/A |
| Discussion | | | |
| Key results | 18 | Summarise key results with reference to study objectives | 8 |
| Limitations | 19 | Discuss limitations of the study, taking into account sources of potential bias or imprecision. Discuss both direction and magnitude of any potential bias | 9 |
| Interpretation | 20 | Give a cautious overall interpretation of results considering objectives, limitations, multiplicity of analyses, results from similar studies, and other relevant evidence | 8-10 |
| Generalisability | 21 | Discuss the generalisability (external validity) of the study results | 12 |
| Other information | | | |
| Funding | 22 | Give the source of funding and the role of the funders for the present study and, if applicable, for the original study on which the present article is based | 13 |

Table S2: Adherence Score to the American Cancer Society/ American Institute Research Fund Score

| **Component** | **Description** | **0=Non adherent** | **1= Modestly adherent** | **2= moderately adherent** | **3= completely adherent** |
| --- | --- | --- | --- | --- | --- |
| 1. Eat at least five - portions/servings of non-starchy vegetables and fruits | One serving= 0.5 cup of vegetables  1 cup of green leafy vegetables | <1 serving | 1-2.99 servings | 3-4.99 servings | ≥5 servings |
| 1. Eat unprocessed cereals and/or pulses (legumes) | Percentage of whole grain compared to total grains and Servings of legumes/day | <30% OR <0.5 servings legumes | 30-39.99% OR  0.50-0.74 servings legumes | 40-49.99% OR 0.75-0.99 servings legumes | ≥50 OR ≥1 servings legumes |
| 1. Limit intake of red and processed meat to less than 500 gms/week | Gms red meat/week and processed meat/day | >840 gms red meat/week OR >84 gms processed meat/day | 672-840 gms red meat/week OR 56-84 gms processed meat/day | 504-671 gms red meat/week OR 28-55.9 gms processed meat/day | <504 gms red meat/week AND <28 gms processed meat/day |
| 1. Consume energy dense foods sparingly | Energy density | ≥225 Kcal/100 gm food OR >14 tsp sugar/day | 176-225 Kcal/100 gm food OR 10.01-14 tsp/day | 126-175 Kcal/ 100 gms food OR 6.01-9.99 tsp/day | <126 Kcal/100 gms AND <6.01 tsp/day |
| 1. Limit alcoholic drinks | Standard drink/day | >2 drinks | 1.01-2 drinks | 0.01-1 | Nondrinkers |
